# Biochar amendment reduces paddy soil nitrogen leaching but increases net global warming potential in Ningxia irrigation, China

**DOI:** 10.1038/s41598-017-01173-w

**Published:** 2017-05-09

**Authors:** Yongsheng Wang, Yansui Liu, Ruliang Liu, Aiping Zhang, Shiqi Yang, Hongyuan Liu, Yang Zhou, Zhengli Yang

**Affiliations:** 1 Key Laboratory of Regional Sustainable Development Modeling, Institute of Geographic Sciences and Natural Resources Research, Chinese Academy of Sciences, Beijing, 100101 China; 2 Ningxia Academy of Agriculture and Forestry Sciences, Yinchuan, 750000 China; 3 Institute of Environment and Sustainable Development in Agriculture, Chinese Academy of Agricultural Sciences/Key Laboratory of Agro-Environment and Climate Change, China Ministry of Agriculture, Beijing, 10081 China; 4 Institute of Environment and Sustainable Development in Agriculture, Chinese Academy of Agricultural Sciences (CAAS), 12 Zhongguancun South Street, Haidian District, Beijing, 100081 China

## Abstract

The efficacy of biochar as an environmentally friendly agent for non-point source and climate change mitigation remains uncertain. Our goal was to test the impact of biochar amendment on paddy rice nitrogen (N) uptake, soil N leaching, and soil CH_4_ and N_2_O fluxes in northwest China. Biochar was applied at four rates (0, 4.5, 9 and13.5 t ha^−1^ yr^−1^). Biochar amendment significantly increased rice N uptake, soil total N concentration and the abundance of soil ammonia-oxidizing archaea (AOA), but it significantly reduced the soil NO_3_^−^-N concentration and soil bulk density. Biochar significantly reduced NO_3_^−^-N and NH_4_^+^-N leaching. The C2 and C3 treatments significantly increased the soil CH_4_ flux and reduced the soil N_2_O flux, leading to significantly increased net global warming potential (GWP). Soil NO_3_^−^-N rather than NH_4_^+^-N was the key integrator of the soil CH_4_ and N_2_O fluxes. Our results indicate that a shift in abundance of the AOA community and increased rice N uptake are closely linked to the reduced soil NO_3_^−^-N concentration under biochar amendment. Furthermore, soil NO_3_^−^-N availability plays an important role in regulating soil inorganic N leaching and net GWP in rice paddies in northwest China.

## Introduction

Synthetic nitrogen (N) fertilizer is currently the largest source of anthropogenic reactive N worldwide^1^ and has enabled the doubling of world food production in the past four decades^2^. However, excessive fertilizer N intended for crops results in environmental pollution problems, such as greenhouse gas (GHG) emissions and surface runoff and leaching^3^. The three main greenhouse gases (i.e., CO_2_, CH_4_, and N_2_O) in combination contribute to more than 90% of anthropogenic climate warming^4^. N leaching may deplete soil fertility, accelerate soil acidification and reduce crop yields^5^.

Global rice paddies occupied more than 1.61 × 10^8^ ha of land and produced 4.82 × 10^8^ T yr^−1^ of grain in 2015~2016, creating a major challenge for N leaching and greenhouse gas emission mitigation^6, 7^. In China, 23% of the nation’s croplands are used for rice production, accounting for approximately 20% of the world’s total^8^. A meta-analysis showed lower N use efficiency of 28.1% during the period 2000–2005 for rice in China^9^, compared with 52% in America and 68% in Europe^10^. The average total N leaching rate was 2.2% in paddy fields^11^. The total amounts of CH_4_ and N_2_O emissions from China’s rice paddies are estimated to be 7.7~8.0 Tg CH_4_ yr^−1^ and 88.0~98.1 Gg N_2_O-N yr^−1^, respectively^12, 13^. Judicious methods are needed to reduce GHG emissions and N leaching losses to achieve lower agricultural environmental costs^14^, while not impairing the capacity of ecosystems to ensure food security.

Biochar is a solid carbon-rich organic material generated by pyrolysis or gasification of biomass residues in the absence of oxygen at a relatively low temperature. Biochar application to agricultural soils has the potential to slow carbon and N release^15, 16^ via the high content of recalcitrant organic carbon in the biochar and concomitant changes in soil properties, which affect microbial activity^17^. Recent reviews have highlighted biochar as a possible method to decrease soil CH_4_ and N_2_O emissions^18, 19^ and reduce N leaching^20^.

The effects of biochar on paddy soil CH_4_ emissions remain controversial depending on the biochar type, climatic conditions and soil properties^21^. A laboratory incubation study showed that amendment with bamboo biochar and rice-straw biochar decreased paddy soil CH_4_ emissions by up to 51% and 91%, respectively^22^, while wheat-straw biochar amendment increased soil CH_4_ emission by 37%^23^. In addition, no significant difference in soil CH_4_ flux was found between a biochar plot and a control plot in Germany^24^. Biochar application can affect N transformation and N fate in soil^16, 25^. The soil N_2_O flux increased significantly in some studies^26, 27^ but substantially decreased or remained unchanged in others^23, 28^. These contrasting results emphasize the need for more studies to assess the role of biochar in mitigating paddy soil CH_4_ and N_2_O fluxes. Moreover, the mechanisms of action are not well understood, which has impeded the adoption of biochar in a wide range of rice ecosystems.

Nitrification, through which microorganisms oxidize ammonium (NH_4_^+^) to create nitrate (NO_3_^−^), releasing N_2_O as a by product, has long been a concern of scientists in paddy soils. Many studies found lower N leaching after biochar amendment in laboratory and field experiments^20, 29^. However, the underlying mechanisms are still controversial. Recent studies have demonstrated that increased water-holding capacity, enhanced microbial biomass and altered bacterial community structure in soils may contribute to the reduction of N leaching^20^. Other studies suggest that reduced N leaching may result from improved plant N use^30, 31^. NH_3_ oxidation to NO_2_^−^, the first and rate-limiting step of nitrification, is catalyzed by ammonia-oxidizing archaea (AOA) and ammonia-oxidizing bacteria (AOB)^32^. Due to the homology of methane monooxygenase and ammonia monooxygenase enzymes, the same habitats, and the variety of analog substrates, CH_4_ in the soil can be simultaneously oxidized by both methanotrophs and ammonia oxidizers^33, 34^. It is essential to explore the links between ammonia oxidizers and the soil CH_4_ and N_2_O fluxes in the field under biochar amendment.

In the upper reaches of the Yellow River basin of China, preliminary studies revealed that biochar amendment significantly improves N use efficiency^35^ and reduces total inorganic N leaching^36^. Consequently, we hypothesized that biochar amendment decreases soil N_2_O emission and reduces NO_3_^−^-N and NH_4_^+^-N leaching. We also hypothesized that biochar amendment increases soil CH_4_ emissions due to the labile carbon input and the positive priming effects of biochar and also increases crop productivity. The aim of the present study was to provide insight into the effects of biochar amendment on paddy soil NO_3_^−^-N and NH_4_^+^-N leaching and soil CH_4_ and N_2_O emissions throughout the entire growth period. By considering the net global warming potential (GWP) of the soil CH_4_ and N_2_O fluxes and the NO_3_^−^-N and NH_4_^+^-N leaching under different treatments, it should be possible to determine the optimal amount of biochar application for China’s rice paddies.

## Materials and Methods

### Study site

This experiment was conducted at Yesheng Town (106°11′35″ E, 38°07′26″ N) in Wuzhong City, China. The temperate continental monsoon climate dominates the region, with a mean temperature of 9.4 °C and a mean annual precipitation of 192.9 mm. The soil is classified as anthropogenic alluvial soil, with a soil texture of 18.25% clay, 53.76% silt, and 27.99% sand. The top soil (0–20 cm) organic matter is 16.1 g kg^−1^, the total N is 1.08 g kg^−1^, and the soil bulk density is 1.33 g cm^−3^.

### Experimental design and rice management

Biochar was applied to the field plots at rates of 0 (C0), 4.5 (C1), 9.0 (C2) and 13.5 t ha^−1^ yr^−1^ (C3). Each treatment was performed in triplicate. A total of 12 plots (30 m × 20 m) were established, and each was separated by plastic film to 130 cm in depth, preventing water interchange between adjacent plots. Each plot was irrigated with an equal amount of water (approximately 14500 m^3^ ha^−1^ yr^−1^). The biochar was produced by pyrolysis of wheat straw at 350–550 °C by the Sanli New Energy Company, Henan Province, China. The biochar had C, N, P and K contents of 65.7%, 0.49%, 0.1% and 1.6%, respectively, with a pH (H_2_O) of 7.78^35^.

Urea was applied at 240 kg N ha^−1^, of which 50% was applied as a base fertilizer before transplanting (26 May, 2014), 30% was applied at the tillering stage (6 June, 2014), and the remaining 20% was applied at the elongation stage (25 June, 2014). Double superphosphate and KCL were also applied as basal fertilizers before transplanting at rates of 90 kg P_2_O_5_ ha^−1^ and 90 kg K_2_O ha^−1^, respectively. Biochar and fertilizers were broadcast on the soil surface and incorporated into the soil by plowing to a depth of approximately 13 cm in May 2014. To maintain consistency, plowing was also performed for the plots without biochar. Rice (Oryza sativa L., cv. 96D10) was sown in a nursery bed on 1 May. Rice seedlings were transplanted on 28 May and harvested on 12 October 2014. Crop management was consistent across plots.

### Measurement of the soil CH_4_ and N_2_O fluxes

The soil CH_4_ and N_2_O fluxes were measured using a static opaque chamber and gas chromatography, as described by Wang *et al*.^33^. Sampling of emitted gases was conducted between 8:00 and 10:00 in the morning. Fluxes were measured twice a week after irrigation and fertilization before the booting stage. Afterwards, the measurement frequency decreased to three times a month during the rice booting, filling and maturity stages and to two times a month during the fallow period. The gas fluxes were measured on 21 occasions during the observation period. The concentrations of CH_4_ and N_2_O in the gas samples were simultaneously analyzed within 24 h using a gas chromatograph (Agilent 7890A, USA) equipped with a flame ionization detector (FID) and an electron capture detector (ECD). High-purity N_2_ and H_2_ were used as the carrier gas and fuel gas, respectively. The ECD and FID were heated to 350 °C and 200 °C, respectively, and the column oven was kept at 55 °C. The CH_4_ and N_2_O fluxes were calculated based on the rate of change in concentration within the chamber, which was estimated as the linear or nonlinear regression slope between concentration and time^37^.

### Soil sampling and analysis

Soil samples (0–20 cm) were collected three times: tillering stage (16 June), filling stage (10 August) and harvest stage (12 October). Five soils from two diagonal lines through each plot were collected and pooled into one composite sample. Soils were sieved to 2-mm mesh size in the field and were then transported to the lab in a biological refrigerator. Soil samples were stored at −80 °C before analysis. Soil NH_4_^+^-N and NO_3_^−^-N were determined using a continuous-flow auto analyzer (Seal AA3, Germany). Soil bulk density was measured using a 100 cm^3^ cylinder. The total N (TN) contents in the bulk soil were determined by dry combustion using the Kjeldahl method^38^.

Soil DNA was extracted from 0.5 g of soil using the Fast DNA®SPIN Kit (Qbiogen Inc., Carlsbad, CA, USA) for soil following the manufacturer’s instructions. The extracted DNA was checked on 1% agarose gel, and the DNA concentration was assessed using a Nanodrop®D-1000 UV-Vis spectrophotometer (NanoDrop Technologies, Wilmington, DE, USA). Tenfold diluted DNA was used in the PCR analysis. Primer pairs *Arch-amoAF/Arch-amoAR* ^39^ and *amoA1F/amoA2R* ^40^ were used for the qPCR of AOA and AOB *amoA* genes, as described by Wang *et al*.^33^. Product specificity was checked by melt curve analysis at the end of the PCR runs and by visualization via agarose gel electrophoresis. A known copy number of plasmid DNA for AOA or AOB was used to generate a standard curve. For all assays, the PCR efficiency was 90–100% and *r* ^2^ was 0.95–0.99.

### Soil leachate sampling and analysis

Soil water samples used for the leaching calculations were collected from lysimeters, as described by Riley *et al*.^41^. Four PPR (polypropylene random) equilibrium-tension lysimeters (ETLs) (0.19 m^2^) were installed at the desired depth (20, 60 and 100 cm) below the soil surface for each treatment condition. Soil leachate samples were collected using 100 ml plastic syringes and were transferred to a plastic tube and stored at 4 °C before analysis. Samples were taken on days 1, 3, 5, 7, and 9 after transplanting and topdressing; subsequent sampling was conducted at 10-day intervals. Soil leachate samples were collected 14 times during the observation period. The NH_4_^+^-N and NO_3_^−^-N leaching losses were calculated by multiplying the N concentration by the leachate volume.

### Rice yield and N uptake

At rice maturity, rice aboveground biomass was estimated by manually harvesting three 0.5 m^2^ areas. Rice straw and grain were oven-dried to a constant weight at 80 °C, weighed, finely ground, sieved, and analyzed for total N using the Kjeldahl method^38^. Total N uptake was calculated from the sum of the N mass in the straw and grain harvested from each plot.

### Statistical analyses

GWP is an index of the cumulative radiative forcing between the present and some chosen later time by a unit mass of gas emitted under specific conditions^42^. To compare net GWP after biochar amendment to soil, we calculated the CO_2_ equivalents for CH_4_ and N_2_O for a time horizon of 100 yr (assuming a GWP of 25 for CH_4_ and 298 for N_2_O) using the following equation.

$${\rm{GWP}}=25\times {{\rm{F}}}_{{{\rm{CH}}}_{4}}+298\times {{\rm{F}}}_{{{\rm{N}}}_{2}{\rm{O}}}$$

Repeated measures of analysis of variance (ANOVA) with the least significant difference (LSD) test were applied to examine the differences in N leaching, soil CH_4_ and N_2_O fluxes, and soil microbial *amoA* gene copy numbers among the different treatments. Biochar amendment was set as a between-subjects factor, and the measurement period was selected as a within-subjects variable. We performed one-way ANOVA with an LSD test to evaluate the effects of biochar amendment on the soil properties, rice yield and N uptake. Linear regression analyses were used to examine the relationships among the soil CH_4_ and N_2_O fluxes, the microbial *amoA* gene copy numbers and the soil inorganic N concentrations. All statistical analyses were conducted using the SPSS software (version 16.0), and differences were considered significant at *P* < 0.05, unless otherwise stated. All figures were drawn using SigmaPlot software (version 10.0).

## Results

### Soil properties, rice yield and N uptake

In the C0 treatment, the average concentration of soil NO_3_^−^-N was 26.52 mg kg^−1^ during the whole observation period (Table 1). The soil NO_3_^−^-N concentration was reduced significantly by 15.10%, 32.13% and 51.02% in the C1, C2 and C3 treatments (Table 1). However, the soil NH_4_^+^-N concentration was only significantly decreased in the C1 treatment(Table 1). Biochar amendment significantly increased soil TN by 10.01~22.22% and significantly decreased bulk density by 4.51~7.81% compared with the treatment without biochar (Table 1). Biochar amendment tended to decrease soil pH (Table 1, *P* > 0.05).

**Table 1:** Soil inorganic N, soil TN, bulk density and soil pH value under the four experimental treatments.

Treatment	NO_3_^−^-N (mg kg^−1^)	NH_4_^+^-N (mg kg^−1^)	TN (g kg^−1^)	Bulk density (g cm^−3^)	Soil pH value
C0	26.52 ± 3.03a	9.81 ± 0.62a	1.08 ± 0.01c	1.33 ± 0.01a	8.62 ± 0.02a
C1	19.14 ± 0.23b	8.44 ± 0.40b	1.15 ± 0.01b	1.28 ± 0.02b	8.58 ± 0.04a
C2	15.30 ± 0.97bc	8.72 ± 0.18ab	1.20 ± 0.02b	1.28 ± 0.02b	8.56 ± 0.03a
C3	12.99 ± 0.39c	9.25 ± 0.32ab	1.32 ± 0.02a	1.27 ± 0.01b	8.56 ± 0.03a

Data are mean ± SE. Lowercase letter in the same column represents significant differences among experimental treatments at the level of 0.05.

**Figure 1:**
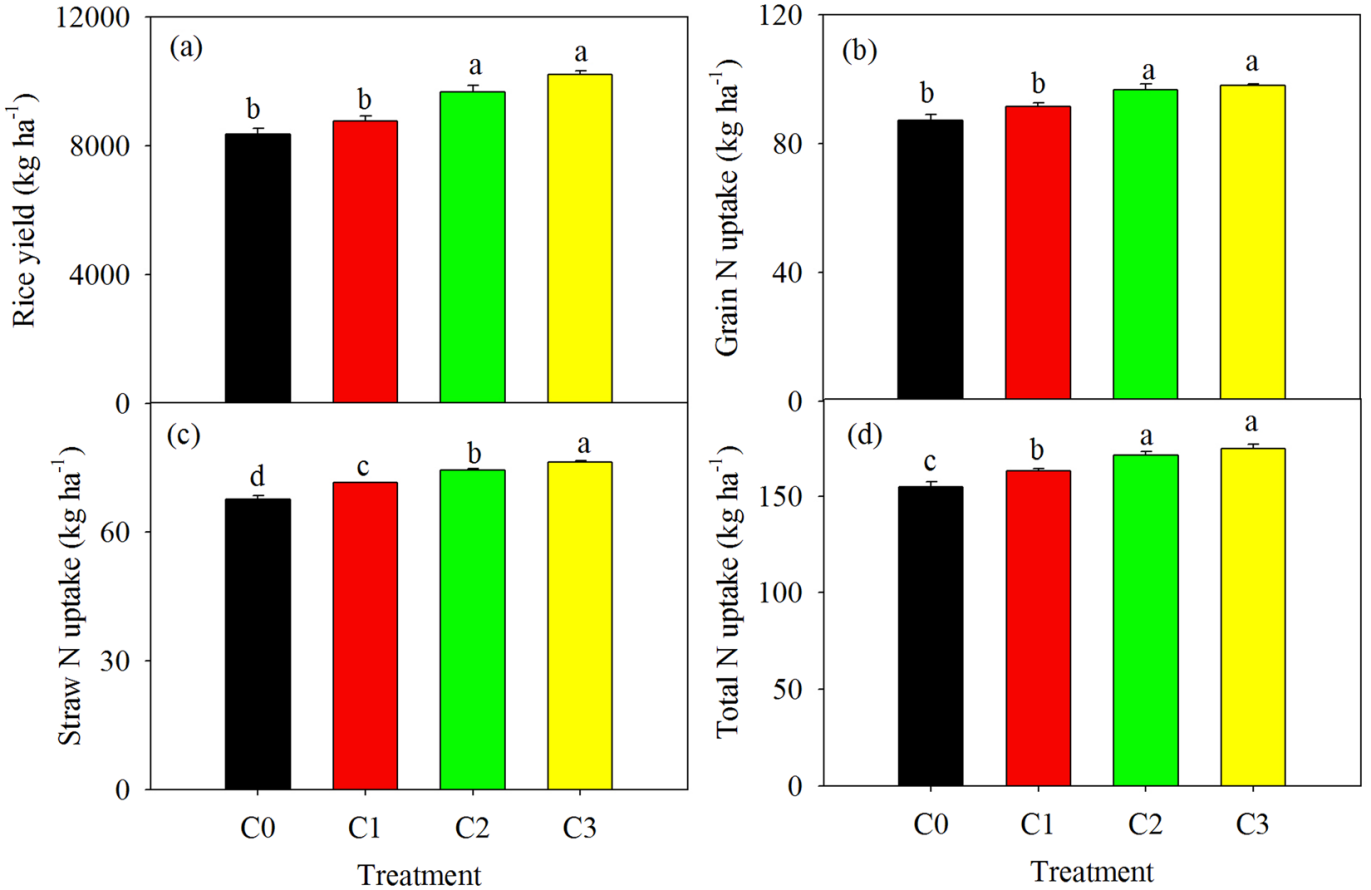
Rice yield (**a**), grain N uptake (**b**), straw N uptake (**c**) and total N uptake (**d**) under the four experimental treatments. Data are shown as means with standard errors. Different letters in the same subfigure indicate significant differences of different treatment according to the LSD test (*P* < 0.05).

In the control, the rice yield and grain N uptake were 8357 kg ha^−1^ and 87.2 kg ha^−1^, respectively. Biochar amendment significantly increased the rice yield and grain N uptake compared with the C0 treatment for the C2 and C3 treatments (Fig. 1a,b). Straw N uptake increased with increasing biochar application rate. Moreover, the relative increase induced by biochar amendment on straw N uptake was 5.62%, 10.06% and 12.87% for the C1, C2 and C3 treatments, respectively (Fig. 1c). In addition, total N uptake was increased by 5.43%, 10.53% and 12.61% in C1, C2 andC3, respectively, compared withC0 (Fig. 1d).

### Soil NO_3_^−^-N and NH_4_^+^-N leaching

**Figure 2:**
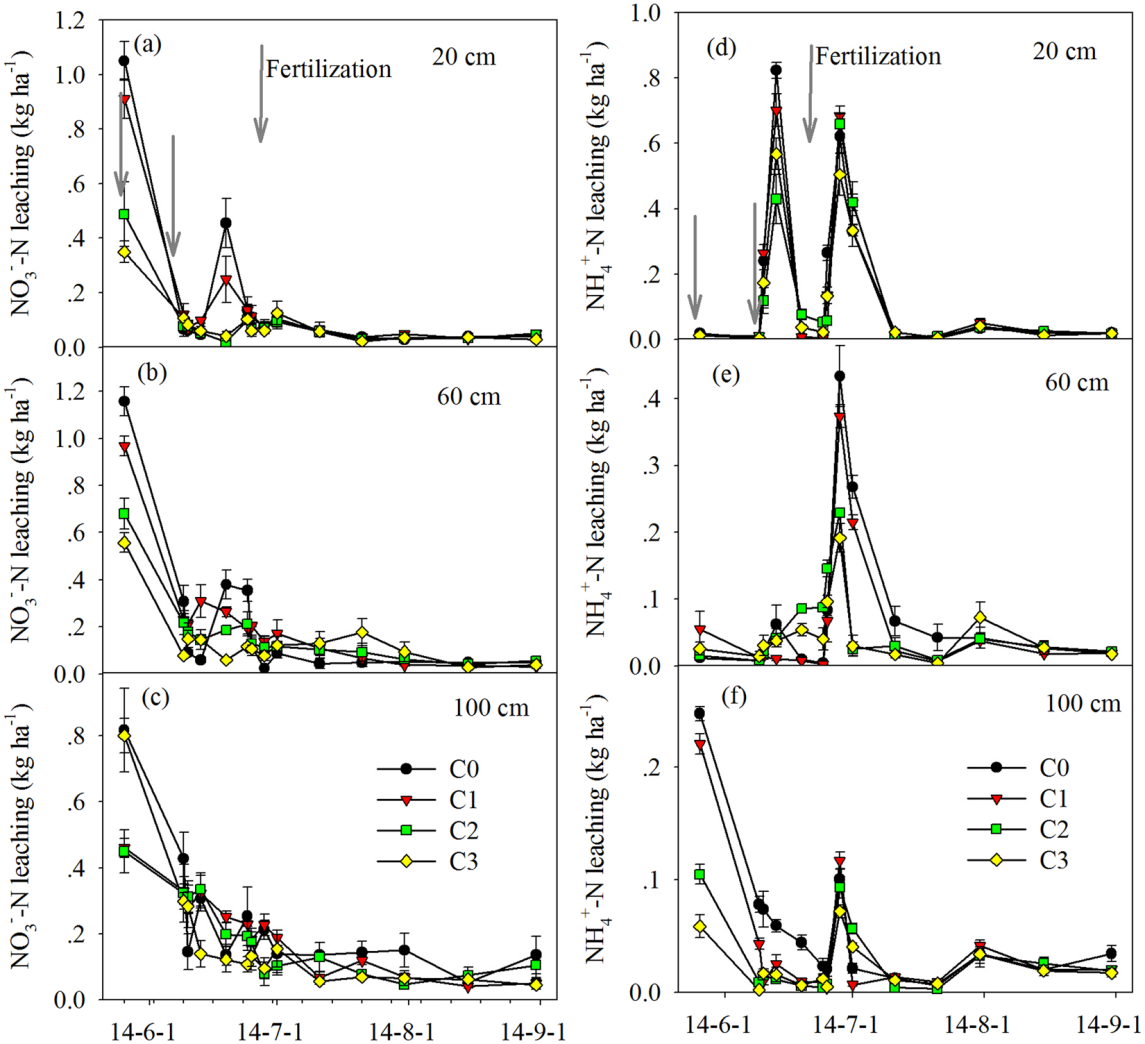
Variation of soil NO_3_^−^-N (**a**–**c**) and NH_4_^+^-N (**d**–**f**) leaching under the four experimental treatments. Data are shown as means with standard errors.

There was a clear seasonal variation in NO_3_^−^-N and NH_4_^+^-N leaching at various soil depths (Fig. 2, Table 2, *P* < 0.001). Supplementary fertilizer N application during the tillering and elongation stages induced NO_3_^−^-N and NH_4_^+^-N leaching peaks. At greater depth, the NO_3_^−^-N and NH_4_^+^-N leaching peaks were dampened. There were no major changes in the NO_3_^−^-N and NH_4_^+^-N leaching during the final two months (Fig. 2). The average NO_3_^−^-N leaching increased with soil depth, whereas NH_4_^+^-N leaching decreased with soil depth (Table 2). Biochar application significantly reduced the mean NO_3_^−^-N and NH_4_^+^-N leaching (Table 2). C1 had significantly decreased NO_3_^−^-N leaching at a depth of 100 cm and NH_4_^+^-N leaching at depths of 60 cm and 100 cm, while C2 and C3 showed significantly decreased NO_3_^−^-N and NH_4_^+^-N leaching throughout the soil profile (Table 2). Furthermore, significant interactions were found between the observation period and biochar treatment, except for the NO_3_^−^-N leaching at 100 cm (Table 2).

**Table 2:** Soil NO_3_^−^-N and NH_4_^+^-N leaching at various soil depths affected by different treatments over the entire experimental period.

Treatments	NO_3_^−^-N (mg L^−1^)			NH_4_^+^-N (mg L^−1^)		
	20 cm	60 cm	100 cm	20 cm	60 cm	100 cm
C0	12.49 ± 0.45a	14.87 ± 0.61a	18.60 ± 0.12a	11.84 ± 0.46a	6.29 ± 0.13a	4.40 ± 0.20a
C1	11.56 ± 0.06a	15.73 ± 0.19a	15.21 ± 0.29b	12.10 ± 0.38a	4.76 ± 0.24b	3.15 ± 0.03b
C2	7.26 ± 0.48b	13.01 ± 0.82b	15.16 ± 1.14b	10.12 ± 0.27b	3.89 ± 0.20c	2.55 ± 0.11c
C3	6.87 ± 0.33b	11.25 ± 0.56b	13.76 ± 0.17b	9.77 ± 0.46b	3.54 ± 0.28c	2.11 ± 0.19c
**ANOVA results**						
Period	<0.001	<0.001	<0.001	<0.001	<0.001	<0.001
Treatment	<0.001	0.003	0.003	0.007	<0.001	<0.001
Period × Treatment	<0.001	<0.001	0.843	0.001	<0.001	<0.001

Data are mean ± SE. Lowercase letter in the same column represents significant differences among experimental treatments at the level of 0.05.

## Soil CH_4_ and N_2_O emissions

**Figure 3:**
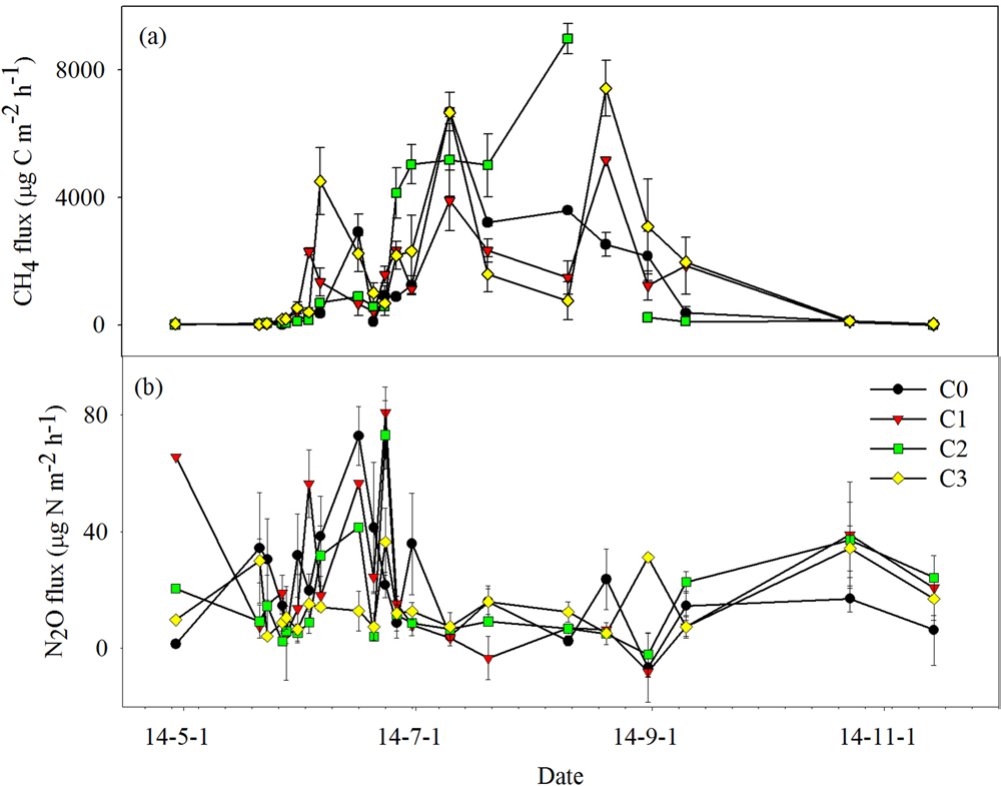
Variation of CH_4_ (**a**) and N_2_O (**b**) fluxes under the four experimental treatments. Data are shown as means with standard errors.

The soil CH_4_ flux significantly varied among growth periods, with the maximum occurring in the booting and filling stages (Fig. 3a, Table 3, *P* < 0.001). In C0, the soil CH_4_ flux ranged from −9.60 µg C m^−2^ h^−1^ to 6688.37 µg C m^−2^ h^−1^, with an average of 1264.54 µg C m^−2^ h^−1^ (Fig. 3a). The cumulative annual soil CH_4_ emission was 110.77 kg C ha^−1^ (Table 3). Biochar amendment significantly increased cumulative soil CH_4_ emissions (Table 3, *P* = 0.008). C2 and C3 showed significantly increased cumulative soil CH_4_ emissions (by 35.16% and 40.62%, respectively) compared with C0 (Table 3). Furthermore, there was a significant interaction for soil CH_4_ flux between observation period and treatment (Table 3, *P* < 0.001).

**Table 3:** Soil CH_4_ and N_2_O fluxes, net GWP, abundances of soil ammonia-oxidizers affected by different treatments over the entire experimental period.

Treatments	CH_4_ (kg C ha^−1^)	N_2_O (kg N ha^−1^)	GWP (kg CO_2_ ha^−1^)	AOA (copies g^−1^ dry soil)	AOB (copies g^−1^ dry soil)
C0	110.77 ± 9.19b	1.87 ± 0.21a	3325.20 ± 261.62c	6.25 ± 0.03c	6.13 ± 0.08a
C1	116.74 ± 5.83b	1.73 ± 0.10ab	3432.92 ± 117.90bc	6.66 ± 0.12b	6.32 ± 0.07a
C2	149.72 ± 10.50a	1.40 ± 0.05b	4160.92 ± 273.83ab	6.54 ± 0.06b	6.24 ± 0.11a
C3	155.76 ± 12.81a	1.33 ± 0.12b	4289.74 ± 341.97a	7.02 ± 0.12a	6.11 ± 0.08a
**ANOVA results**					
Period	<0.001	<0.001		0.074	0.011
Treatment	0.008	0.039		<0.001	0.349
Period × Treatment	<0.001	<0.001		0.009	0.684

Data are mean ± SE. Lowercase letter in the same column represents significant differences among experimental treatments at the level of 0.05.

Soil N_2_O flux showed an obvious variation among growth stages (Fig. 3b, Table 3, *P* < 0.001). The maximum soil N_2_O emission occurred during the rice tillering stage (Fig. 3b). Soil N_2_O flux fluctuated from 2.55 µg N m^−2^ h^−1^ to 72.70 µg N m^−2^ h^−1^inC0, with an average of 63.88 µg N m^−2^ h^−1^ (Fig. 3b). This rate translated into a cumulative annual soil N_2_O emission of 1.87 kg N ha^−1^ (Table 3). Biochar amendment significantly reduced soil N_2_O emissions (Table 3, *P* = 0.039), and the interaction between observation period and treatment was also significant (Table 3, *P* < 0.001). C2 and C3 showed significantly decreased annual cumulative N_2_O emissions (by 25.13% and 28.88%, respectively, relative to C0) (Table 3). However, the C1 treatment decreased soil N_2_O emissions (Table 3).

Biochar amendment consistently increased the net GWP, and C2 and C3 showed the largest increases (29.01% and 25.13%, respectively) (Table 3). However, the increase in net GWP elicited by the C1 treatment was only 3.24% (Table 3).

### Abundance of soil AOA and AOB communities

**Figure 4:**
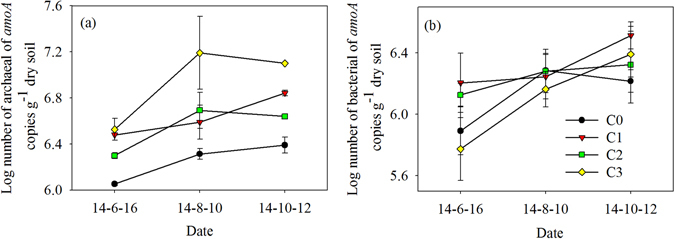
Variation of AOA (**a**) and AOB (**b**) *amoA* gene copy numbers and arithmetic mean under the four experimental treatments. Data are shown as means with standard errors.

Soil AOA *amoA* copy numbers exhibited a slight seasonal variation (Fig. 4a, Table 3, *P* = 0.074), while soil AOB *amoA* copy numbers showed a clear pattern of seasonal changes (Fig. 4b, Table 3, *P* = 0.011). Biochar amendment significantly increased soil AOA *amoA* copy numbers (Table 3, *P* < 0.001). The highest AOA *amoA* gene copy numbers were observed in the C3 treatment(12.32% greater than that of C0) (Table 3). Conversely, there was no significant difference in soil AOB *amoA* copy numbers among the four treatment (Table 3, *P* = 0.349).

### Relationships between soil fluxes and ammonia-oxidizer abundances and soil inorganic N concentrations

The soil CH_4_ fluxes were negatively linearly correlated with the soil NO_3_^−^-N concentration (Table 4). The soil N_2_O fluxes were negatively correlated with the soil AOA abundance and positively correlated with the soil NO_3_^−^-N concentration (Table 4). The soil AOA abundance was negatively correlated with the soil NO_3_^−^-N concentration, whereas the soil AOB abundance was not correlated with any of the measured soil fluxes or inorganic N concentrations (Table 4).

**Table 4:** Correlation coefficients (R^2^) for the relationships among soil CH_4_ and N_2_O fluxes, soil ammonia-oxidizers, soil NO_3_^−^-N and NH_4_^+^-N concentrations.

	CH_4_	N_2_O	AOA	AOB
AOA	0.24	0.43(−)*		
AOB	0.04	0.06	—	—
NO_3_^−^-N	0.45(−)*	0.65(+)**	0.59(−)**	0.07
NH_4_^+^-N	0.04	0.18	0.02	0.01

Note: Significance: *P < 0.05; **P < 0.01. For all correlations, n = 12. (+), positive relationship; (−), negative relationship.

## Discussion

### Effects of biochar amendment on soil NO_3_^−^-N and NH_4_^+^-N leaching

**Figure 5:**
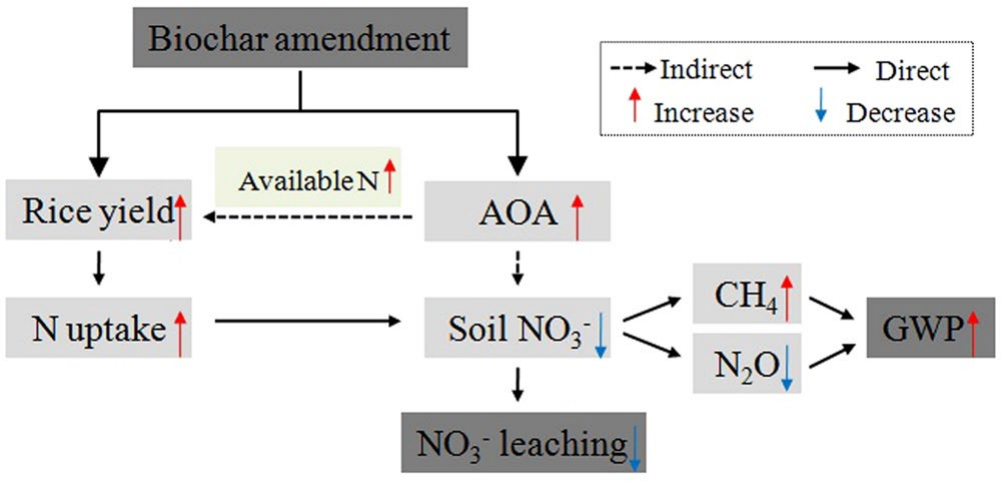
Potential mechanisms of paddy soil N leaching and total GWP in response to biochar amendment.

In our study, significant reductions in soil NO_3_^−^-N and NH_4_^+^-N leaching were observed in the C2 and C3 treatment conditions, while the significant decreases in the C1 treatment were NO_3_^−^-N leaching at the depth of 100 cm and NH_4_^+^-N leaching at depths of 60 cm and 100 cm (Table 2). These results confirmed our first hypothesis that biochar amendment reduces soil NO_3_^−^-N and NH_4_^+^-N leaching. Increases in N retention or absorption in soil^29, 43^ and stimulation of crop N uptake^44^ have generally been hypothesized to be the primary causes of reduced N leaching after biochar application. In our study, biochar amendment significantly increased the total soil N concentration and rice yield (Table 1), consistent with the meta-analysis results reported by Biederman and Harpole^45^. Furthermore, biochar amendment increased AOA activity (Table 4), producing more available N for crop growth and increased rice N uptake (Fig. 5). The reduced soil NO_3_^−^-N and NH_4_^+^-N concentrations decreased the inorganic N pool for leaching (Table 1). Therefore, the reduced soil NO_3_^−^-N and NH_4_^+^-N concentrations induced by biochar application may be the main cause of the reduction in NO_3_^−^-N and NH_4_^+^-N leaching (Fig. 5). In addition, the increased soil water holding capacity due to the reduced soil bulk density (Table 1) may have also reduced NO_3_^−^-N and NH_4_^+^-N leaching^46^.

### Effects of biochar amendment on the soil CH_4_ and N_2_O fluxes

The net CH_4_ flux from soil is the sum of production and oxidation. The effects of biochar amendment on the CH_4_ flux were thus unclear. In agreement with previous studies^24, 47^, the application of biochar at rates of 9 t ha^−1^ yr^−1^ and 13.5 t ha^−1^ yr^−1^ significantly increased the soil CH_4_ flux by 35.16% and 40.62%, respectively (Table 3). The promotion of the soil CH_4_ flux was comparable to that of the Tai Lake plain in China for biochar amendment at rates of 10 t ha^−1^and 40 t kg ha^−1^ ^23^. This was attributed to the following three aspects. First, the soil NH_4_^+^-N accumulation decreased soil CH_4_ oxidation by altering the activity and composition of the methanotrophic community^48^. However, biochar amendment did not significantly change the soil NH_4_^+^-N accumulation (Table 1), and there was no significant relationship between the soil CH_4_ flux and soil NH_4_^+^-N concentration (Table 4). Wang *et al*.^49^ found that soil NO_3_^−^-N accumulation could significantly promote soil CH_4_ uptake. The lower soil CH_4_ uptake was due to the decreased soil NO_3_^−^-N concentration under biochar amendment (Table 1), which increased the soil CH_4_ emissions in our study (Table 3, Fig. 5). The significant negative relationship between the soil NO_3_^−^-N concentration and the soil CH_4_ flux reflected this prediction (Table 4). Second, methanotrophs use the sorbed organic compounds in addition to CH_4_ because methanotrophs can utilize a variety of substrates^50^. Therefore, biochar amendment reduced the net soil CH_4_ oxidation^51^. Third, Knoblauch *et al*.^52^ reported that the labile components of biochar increase the substrate supply and create a favorable environment for methanogens^53^. The lower pH in our biochar plots may have promoted methanogenic archaea, which have an optimal pH of 7^54^. Thus, a larger archaeal population may temporarily have increased CH_4_ emissions in the biochar treatment^55^ until the emissions declined due to the oxic environment. However, because we only measured the net effects, we could not distinguish between reduced soil methanotrophic activity and increased methanogenic activity in this study.

Suppression of soil N_2_O emissions following biochar amendment has been observed both under laboratory conditions^25, 26^ and in the field^23, 47^. Enhanced soil aeration^27^, altered ammonia-oxidizer and denitrifier activity^56^, sorption of NH_4_^+^-N or NO_3_^−^-N by biochar^12^ and the presence of inhibitory compounds such as ethylene^57^ have been suggested as mechanisms to explain the reduction inN_2_O flux with biochar amendment. In anaerobic paddy soil, N_2_O production from denitrification is thought to be the dominant source. Baggs^58^ suggested that decreased total N denitrification and enhanced reduction of N_2_O to N_2_ can lead to lower N_2_O denitrification in soil. A reduction in NO_3_^−^-N availability would decrease the total denitrified N and would reduce the ratio of N_2_O/(N_2_ + N_2_O)^17^. In our study, biochar amendment reduced the soil NO_3_^−^-N concentration by accelerating ammonia oxidation (Fig. 4, Table 3) and promoting total N uptake in rice (Fig. 1). The significant negative correlation between the soil NO_3_^−^-N concentration and soil AOA abundance provided direct evidence for this result (Table 4). Soil NO_3_^−^-N availability was positively correlated with soil N_2_O flux (Table 4), which could partially explain the reduction in soil N_2_O flux in the studied paddy soil (Fig. 5). In addition, evidence for the decreased N_2_O denitrification was provided by the decreased soil bulk density after biochar amendment (Table 1). Soil pH was not significantly changed after biochar amendment at our study site (Table 1). However, soil Eh was not monitored when the gas samples were taken *in situ*. Mechanisms associated with the oxidation and reduction of nitrogen species need to be studied under the alternating redox conditions on the surface of biochar.

However, our flux results were drawn from relatively few measurements. The gas fluxes were measured on 19 occasions during 150 days of rice growth, with an average sampling interval of 8 days. This frequency was slightly lower than the average 6.5 day interval of the reviewed rice paddy studies in China with single biochar amendment and biochar combined with NPK compound fertilizer (Supplementary Table S1). The effects of biochar amendment on the soil CH_4_ flux are contradictory, including positive, negative, and neutral effects. Moreover, biochar amendment is mainly reported to decrease soil N_2_O flux. Increased soil CH_4_ flux and decreased soil N_2_O flux were observed in our study. This evidence suggests that our conclusions can be drawn from relatively few measurements.

Biochar is considered to be an effective tool to mitigate GWP via carbon sequestration in soil and to influence carbon mineralization through priming effects. In the present study, the changes in net GWP were not significant between C1 and C0 treatments, while biochar amendment significantly increased paddy soil CH_4_ emissions and decreased N_2_O emissions in the C2 and C3 treatments, leading to a significantly increased net GWP (Table 3). This result is in line with that of a rice paddy from Tai Lake plain, China^23^. Our results indicate that higher amounts of biochar amendment are associated with a risk of increased paddy net GWP in northwest China (Fig. 5). The stimulation effects of biochar on native soil organic carbon mineralization decreases with time due to the depletion of labile SOC from the initial positive priming and stabilization of native SOC via biochar-induced organ-mineral interactions during 5-year laboratory incubations^59^. In another 120-day incubation study, the time effects showed an initial increase followed by a decrease and stabilization, resulting in a significantly increased sequestration of carbon in the soil over the long term compared with conventional biowaste amendments^60^. In our study, improved rice yield and reduced soil nitrous oxide emissions contributed to the mitigation potential of biochar amendment. However, the response of native soil organic carbon mineralization to biochar amendment remains uncertain. Relative priming effects and cumulative CO_2_ emissions studies are needed to evaluate the GWP of biochar amendment in our paddy soil.

## Conclusions

The effects of biochar amendment on soil N leaching and soil CH_4_ and N_2_O fluxes were investigated in paddy soil in northwest China. We found that biochar amendment significantly decreased soil NO_3_^−^-N and NH_4_^+^-N leaching, but that the C2 and C3 treatments significantly increased soil CH_4_ emissions and reduced N_2_O emissions, leading to significantly increased net GWP. Biochar amendment significantly increased soil AOA abundance and rice N uptake. Soil NO_3_^−^-N availability can explain the responses of soil N leaching and soil CH_4_ and N_2_O fluxes to biochar amendment. Our results indicated are commended dose of biochar amendment of 4.5 t ha^−1^ yr^−1^ with conventional N application in the study area. The responses of the soil CH_4_ and N_2_O fluxes to biochar amendment were also influenced by the interannual variability in climate, temperature and precipitation. The long-term effects of biochar amendment on N leaching and net GWP in rice production required further investigation to identify the most cost-effective and environmentally friendly management practices for rice culture.

## Electronic supplementary material

Supplementary table
