# Supplementary material for: Biochar amendment reduces paddy soil nitrogen leaching but increases net global warming potential in Ningxia irrigation, China
Source: Sci Rep. 2017 May 9;7:1592. doi: 10.1038/s41598-017-01173-w (PMC5431622; doi:10.1038/s41598-017-01173-w)
Supplement: Supplementary file 1 — Supplementary table [file 41598_2017_1173_MOESM1_ESM.doc]

**Biochar amendment reduces paddy soil nitrogen leaching but increases net global warming potential in Ningxia irrigation, China**

Yongsheng Wang^1^, Yansui Liu^1^, Ruliang Liu^2^, Aiping Zhang^3^*, Shiqi Yang^3^, Hongyuan Liu^3^, Yang Zhou^1^, Zhengli Yang^3^

*^1^ Key Laboratory of Regional Sustainable Development Modeling, Institute of Geographic Sciences and Natural Resources Research,* *Chinese Academy of Sciences, Beijing 100101,China 
^2^* *Ningxia Academy of Agriculture and Forestry Sciences, Yinchuan 750000, China*

*^3^* *Institute of Environment and Sustainable Development in Agriculture, Chinese Academy of Agricultural Sciences/**Key Laboratory of Agro-Environment and Climate Change, China Ministry of Agriculture, Beijing 10081, China*

**Type of contribution:** Research article

**Running title:** Biochar amendment reduces nitrogen leaching but increases net GWP

**Manuscript submitted to** *Scientific Reports*

**Date of preparation:** December 5, 2016

**Manuscript information:** 29 pages, 4 tables, and 5 figures

**Correspondence:** Dr. Aiping Zhang, tel.+86-10-82108793, E-mail: apzhang0601@126.com

**Present address:** 11A Datun Road, Chaoyang District, Beijing 100101, China

Table S1 Review of studies carried out in China with both straight biochar and biochar NPK compound

| Crop type | Trial | Sampling  method | Duration | Frequency  (Time) | Results | |
| --- | --- | --- | --- | --- | --- | --- |
|  |  |  |  |  | CH_4_ | N_2_O |
| Rice^1^ | Field | Chamber | 6 months | 6 in elongation stage, 6 in milk-ripe stage, total of 12 occasions | Decreased | Decreased |
| Rice^2^ | Field | Chamber | 100 days | 3-4 day interval after transplanting, total of 17 occasions | Increased | Decreased |
| Rice^3^ | Field | Chamber | 4 months | 3-day interval in pre-growing stage and decreased frequency in drying period, total of 26 occasions | Decreased | Decreased |
| Rice^4^ | Field | Chamber | 14 months | 10-day interval, total of 10 occasions | Decreased | Decreased |
| Rice^5^ | Incubation | NaHCO_3_ solution drainage | 7 weeks | 1, 4, 7, 14, 28 and 49 day after incubation, total of 6 occasions | Decreased | No data |
| Rice^6^ | Incubation | Chamber | 16 weeks | Once a week in pre-growing stage and 10-15 day interval in later-growing stage, total of 11 occasions | Decreased | Decreased |
| Rice-wheat^7^ | Field | Chamber | 132 days | Once a week, total of 11 occasions | Decreased in combined N fertilization, increased in single biochar amendment | Decreased |
| Rice^8^ | Incubation | Chamber | 120 days | 3 days interval, total of 19 occasions | No data | No change in Liaoning Province, increased in Shandong, Jiangsu and Zhejiang Provinces |
| Rice^9^ | Field | Chamber | 90 days | 10-day interval, total of 10 occasions | Decreased | No data |
| Rice^10^ | Field | Chamber | 15 weeks | 10-day interval, total of 10 occasions | Decreased | Decreased |
| Rice^11^ | Field | Chamber | 15 weeks | 10-day interval, total of 12 occasions | Decreased | Decreased |
| Double rice^12^ | Field | Chamber | 74 weeks | 2-day interval before drying period, 4 days interval after re-watering, total of 71 occasions | Decreased | Decreased |
| Rice^13^ | Field | Chamber | 113 days | Once a week, total of 16 occasions | Increased | Decreased |
| Rice^14^ | Field | Chamber | 40 weeks | Once a week | Increased in the first year, no change in the second year | Decreased |
| Rice^15^ | Field | Chamber | One season | Once a week | No data | Decreased |
| Rice^16^ | Field | Chamber | 120 days | 10-day interval, total of 12 occasions | Increased | Decreased |
| Rice^17^ | Incubation | Headspace air sampling | 60 days | 1, 2, 3, 4, 5, 6,7, 10, 11, 15, 17, 18, 20, 23, 25, 30, 35, 40, 45, 50, 55 days after incubation, total of 20 occasions | No data | Decreased |
| Rice^18^ | Incubation | Headspace air sampled | 60 days | 1, 2, 3, 4, 5, 6,7, 10, 11, 15, 17, 18, 20, 23, 25, 30, 35, 40, 45, 50, 55 days after incubation, total of 20 occasions | Decreased | Decreased |
| Rice^19^ | Field | Chamber | 120 days | Total of 20 occasions | No change | No change in the acidic Ultisol, increased in slightly alkaline Inceptisol |
| Rice^20^ | Field | Chamber | 116 days | 6-14 day interval, total of 13 occasions | Decreased |  |
| Rice^21^ | Incubation | Headspace air sampling | 49 days | 1, 4, 7, 14, 28 and 49 days after biochar addition, total of 6 occasions | Decreased |  |
| Double rice^22^ | Field | Chamber | 7 months | 2-day interval from transplanting to drying period, 3 days interval after drying period, total of 79 occasions | Decreased | Decreased |
| Rice-wheat^23^ | Field | Chamber | 9 months | Once a week, intensified sampling in fertilization, total of 52 occasions | No change in the rate of 20 t ha^-1^, decreased in the rate of 40 t ha^-1^ | Decreased |
| **Average** | | | **145 days** | **total of 22 occasions** |  |  |

**Supplementary References**

1 Wang, M. Y., Xu, X. P., Wang, G. L., Su, C. J. & An, W. L. Effect of slag and biochar amendment on greenhouse gases emissions and related microorganisms in paddy fields. *Acta Scientiae Circumstantiae*, doi:10.13671/j.hjkxxb.2016.0278 (2016).

2 Peng, H., Ji, X., Wu, J., Tian, F. & Huo, L. Integrated effect of decreasing CH_4_ and N_2_O emission by Biochar incorported to paddy field on late rice. *Ecology & Environmental Sciences* **20**, 1620-1625, doi:10.16258/j.cnki.1674-5906.2011.11.016 (2011).

3 Meng, M. *et al.* Effect of biochar on CH_4_ and N_2_O emissions from early rice field in South China. *Chinese Journal of Agrometeorology* **34**, 396-402, doi:10.3969/j.issn.1000-6362.2013.04.004 (2013).

4 Liu, Y. X. *et al.* Effects of biochar application on greenhouse gas emission from paddy soil and its physical and chemical properties. *Chinese Journal of Applied Ecology* **24**, 2166-2172 (2013).

5 Liu, Y. X. *Effect of biochar on the characteristic of nitrogen loss and greenhouse gas emission from soil*, Zhejiang University, Hangzhou, (2011).

6 Wang, X. X. *et al.* Effects of bamboo biochar amendments on methane and nitrous oxide emission from paddy soil. *Journal of Agro-Environment Science* **33**, 198-204, doi:10.11654/jaes.2014.01.026 (2014).

7 Zhou, Z. Q., Li, L., Zhang, H. & Xiong, Z. Q. Effects of wheat straw biochar and nitrogen amendment on methane and nitrous oxide distribution characteristics within soil profile in rice-wheat annual rotations. *Journal of Nanjing Agricultural University* **38**, 431-438, doi:10.7685/j.issn.1000-2030.2015.03.012 (2015).

8 Pan, Y. F. *Influence of biochar on ammonia oxidation microbial community in paddy soil*, Zhejiang University, Hangzhou, (2014).

9 Jiang, C. *et al.* Effect of biochar returning to paddy field on CH_4_ emission reduction. *Transactions of the Chinese Society of Agricultural Engineering* **29**, 184-191, doi:10.3969/j.issn.1002-6819.2013.15.022 (2013).

10 Liu, Y. X. *et al.* Effects of biochar application on greenhouse gas emission from paddy soil and its physical and chemical properties. *Chinese Journal of Applied Ecology* **24**, 2166 (2013).

11 Li, S., Li, H., Fang, X. & Shi, H. Biochar input to reduce trace greenhouse gas emission in paddy field. *Transactions of the Chinese Society of Agricultural Engineering* **30**, 234-240, doi:10.3969/j.issn.1002-6819.2014.21.028 (2014).

12 Qin, X. *et al.* Impact of biochar amendment on carbon emissions intensity in double rice field in South China. *Transactions of the Chinese Society of Agricultural Engineering* **31**, 226-234, doi:10.3969/j.issn.1002-6819.2015.05.032 (2015).

13 Zhang, B. Consistent effect of biochar amendment on soil quality rice yield and greenhouse gas emissions, Nanjing Agriculture University, Nanjing, (2012).

14 Zhang, B. *et al.* Changes in soil properties, yield and trace gas emission from a paddy after biochar amendment in two consecutive rice growing cycles. *Scientia Agricultura Sinica* **45**, 4844-4853, doi:10.3864/j.issn.0578-1752.2012.23.011 (2012).

15 Liu, X. Y. *et al.* Can biochar amendment be an ecological engineering technology to depress N_2_O emission in rice paddies?—A cross site field experiment from South China. *Ecol Eng* **42**, 168-173 (2012).

16 Zhang, A. F. *et al.* Effect of biochar amendment on yield and methane and nitrous oxide emissions from a rice paddy from Tai Lake plain, China. *Agr Ecosyst Environ* **139**, 469-475, doi:10.1016/j.agee.2010.09.003 (2010).

17 Wang, J., Zhang, M., Xiong, Z., Liu, P. & Pan, G. Effects of biochar addition on N_2_O and CO_2_ emissions from two paddy soils. *Biology and Fertility of Soils* **47**, 887-896 (2011).

18 Wang, J., Pan, X., Liu, Y., Zhang, X. & Xiong, Z. Effects of biochar amendment in two soils on greenhouse gas emissions and crop production. *Plant Soil* **360**, 287-298, doi:10.1007/s11104-012-1250-3 (2012).

19 Xie, Z. B. *et al.* Impact of biochar application on nitrogen nutrition of rice greenhouse-gas emissions and soil organic carbon dynamic in two paddies soils of China. *Plant Soil* **370**, 527-540, doi:10.1007/s1104-013-1636-x (2013).

20 Feng, Y. Z., Xu, Y. P., Yu, Y. C., Xie, Z. B. & Lin, X. G. Mechanisms of biochar decreasing methan emission from Chinese paddy soils. *Soil Biol Biochem* **46**, 80-88, doi:10.1016/j.soilbio.2011.11.016 (2012).

21 Liu, Y. X. *et al.* Reducing CH_4_ and CO_2_ emissions from waterloged paddy soil with biochar. *J Soil Sediment* **11**, 930-939, doi:10.1007/s11368-011-0376-x (2011).

22 Zhang, W. H. *The impact of diffeent straw returning forms and rice varieties on CH_4_ and N_2_O emission in a double rice field*. Chinese Academy of Agriculture Science, Beijing, (2016).

23 Li, L., Zhou, Z. Q., Pan, X. J. & Xiong, Z. Q. Rice-wheat rotation during rice and wheat growing seasons relative to timing of amendment. Acta Pedologica Sinica 52, 839-848, doi:10.11766/trxb201407160357 (2015).
